# Combined HDAC1 and HDAC2 Depletion Promotes Retinal Ganglion Cell Survival After Injury Through Reduction of p53 Target Gene Expression

**DOI:** 10.1177/1759091415593066

**Published:** 2015-06-27

**Authors:** Frédéric Lebrun-Julien, Ueli Suter

**Affiliations:** 1 Institute of Molecular Health Sciences, Department of Biology, Swiss Federal Institute of Technology (ETH) Zürich, CH, Switzerland

**Keywords:** axotomy, central nervous system, histones deacetylases, neuroprotection, p53, retinal ganglion cell

## Abstract

Histones deacetylases (HDACs), besides their function as epigenetic regulators, deacetylate and critically regulate the activity of nonhistone targets. In particular, HDACs control partially the proapoptotic activity of p53 by balancing its acetylation state. HDAC inhibitors have revealed neuroprotective properties in different models, but the exact mechanisms of action remain poorly understood. We have generated a conditional knockout mouse model targeting retinal ganglion cells (RGCs) to investigate specifically the functional role of HDAC1 and HDAC2 in an acute model of optic nerve injury. Our results demonstrate that combined HDAC1 and HDAC2 ablation promotes survival of axotomized RGCs. Based on global gene expression analyses, we identified the p53-PUMA apoptosis-inducing axis to be strongly activated in axotomized mouse RGCs. Specific HDAC1/2 ablation inhibited this apoptotic pathway by impairing the crucial acetylation status of p53 and reducing PUMA expression, thereby contributing to the ensuing enhanced neuroprotection due to HDAC1/2 depletion. HDAC1/2 inhibition and the affected downstream signaling components emerge as specific targets for developing therapeutic strategies in neuroprotection.

## Introduction

Lysine acetylation is an essential, reversible, and highly regulated posttranslational modification. This crucial process is controlled by histone acetyltranferase (HATs) and histone deacetyltransferase (HDACs) that are together responsible for the critical balance of acetylation and deacetylation within the cell. In its epigenetic function, HDAC activity triggers condensed chromatin architecture that limits transcription by removing acetyl groups from histones. However, HDAC scan also contribute to nonepigenetic regulation in that HDACs are capable of deacetylating and centrally regulating the activity of several transcription factors as well as of other proteins (Glozak et al., 2005; Yao and Yang, 2011).

HDACs have emerged as promising targets for therapeutic interventions in pathological conditions, including neurodegenerative disorders, and HDAC inhibitors have been shown to rescue or delay neuronal death (Chuang et al., 2009; Dietz and Casaccia, 2010; Zalewska et al., 2014). In particular, in the optic system, HDAC3 appears to play a major regulatory role in injured retinal ganglion cells (RGCs) and in a glaucoma model (Pelzel et al., 2012; Chindasub et al., 2013).

The tumor suppressor p53, an essential transcription factor for genes involved in cell cycle progression and initiation of apoptosis, especially also in neurological insults (Morrison et al., 2003), is a nonhistone protein target of HDACs (Gu and Roeder, 1997). Indeed, p53 can be acetylated by p300/CBP at multiple lysine residues within the C-terminal DNA binding regulatory domain, strongly correlated with protein stabilization and activation (Brooks and Gu, 2011). On the other hand, HDAC1, HDAC2, or SIRT1, a member of the Class III family of HDACs, can act as p53 deacetylases (Murphy et al., 1999; Juan et al., 2000; Luo et al., 2000, 2001; Harms and Chen, 2007; LeBoeuf et al., 2010). Deacetylation of p53 by HDACs is commonly accepted to reduce its transcriptional activity, although the acetylation-dependent activity of p53 appears to be acetylation-site specific and cell-type specific (Brochier et al., 2013).

The mechanisms by which HDAC inhibitors are neuroprotective are only partially understood. In addition, most of the available studies are based on drug-mediated inhibition with the intrinsic caveat of specificity problems. In this study, we have used specific genetic depletion of both HDAC1 and HDAC2 (HDAC1/2) in RGCs *in vivo* to avoid misguiding off-target effects. Using the optic nerve transection model to induce RGC apoptosis, we found a pronounced neuroprotective effect due to HDAC1/2 depletion. Furthermore, we discovered that the p53-PUMA apoptosis-inducing axis was strongly activated in axotomized RGCs of control mice, reminiscent of reports in the rat (Wilson et al., 2013, 2014). HDAC1/2 ablation inhibited this proapoptotic function of p53, consistent with its aberrant acetylation status and impaired expression of the proapoptotic factor PUMA.

## Materials and Methods

### Mouse Mutants

Mice homozygous for *Hdac1*^loxP/loxP^ and *Hdac2*^loxP/loxP^ alleles (Yamaguchi et al., 2010) were crossed with mice expressing a tamoxifen-inducible form of Cre recombinase under control of the *Thy-1* gene regulatory sequences (*Thy-1-YFP*: *Thy-1-CreERT2*, *SLICK-H*; Young et al., 2008) to ablate HDAC1 and HDAC2 in neurons. Genotypes were determined by polymerase chain reaction (PCR) using genomic DNA. Animal use was approved by the veterinary office of the Canton of Zurich, Switzerland.

### Retrograde Labeling and Quantification of Neuronal Survival

Retrograde labeling of RGCs was performed using Fluorogold (FG; 2%, Fluorochrome) in 0.9% NaCl, which was applied to both superior colliculi as described (Sapieha et al., 2005). The left optic nerve was transected 0.5 to 1 mm from the optic nerve head at 1 week after FG application as previously described (Berkelaar et al., 1994; Cheng et al., 2002; Lebrun-Julien et al., 2010). Mice were perfused with 4% paraformaldehyde; eyes were dissected and flat mounted vitreal side up on glass slides. FG-positive RGCs were counted in 12 retinal zones: Three areas in each eye quadrant (dorsal, ventral, nasal, and temporal) were examined, corresponding to a total area of 0.5 mm^2^. Wild-type uninjured retinas were compared with control (*Cre***^−^**; *Hdac1*^loxP/loxP^, and *Hdac2*^loxP/loxP^) and HDAC1/2 mutant uninjured or axotomized retinas.

### Retinal Immunohistochemistry

Mice were killed by intraperitoneal injection injection of pentobarbital (150 mg/kg; Nembutal, Abbott Laboratories) and perfused with 4% paraformaldehyde. Tissue sections were incubated in 5% bovine serum albumin and 0.3% Triton X-100 (Sigma) to block nonspecific binding and then incubated with primary antibodies overnight at 4℃, followed by incubation with secondary antibodies at room temperature. Antibodies include anti-anti-GFP (Aves labs, GFP-1020), anti-HDAC1 (PA1-860, Thermo Scientific), anti-HDAC2 (H2663, Sigma), anti-Phospho-p53 Ser15 (9284, Cell Signaling), and anti-PUMA (7467, Cell Signaling). After washing with phosphate-buffered saline, sections were incubated with secondary antibodies coupled to Alexa 488 (1:250, Invitrogen) or Cy3 (1:250, Jackson Laboratories) for 1 hr at room temperature, washed in phosphate-buffered saline, and mounted with IMMU-MOUNT (Thermo Scientific). Staining was observed using a fluorescence microscope (Axioplan2 Imaging, Carl Zeiss). Images were digitized with a Powershot G5 camera (Canon) and acquired with Axio Vision 4.5 software (Carl Zeiss). Images were further processed (levels adjusted) using Photoshop CS5 software (Adobe).

### Western Blot

Retinas were homogenized in lysis buffer (20 mM Tris [pH 8.0], 135 mM NaCl, 1% sodium dodecyl sulfate [SDS], and 10% glycerol supplemented with protease inhibitors) and centrifuged at 14,000 rpm for 5 min. The supernatants were collected and diluted in Laemmli sample buffer 5× (4% SDS, 10% glycerol, 0.004% bromophenol blue, 0.1 M dithiothreitol, and 0.125 M Tris, pH 6.8). Extracts were processed using standard sodium dodecyl sulfate polyacrylamide gel electrophoresis (SDS-PAGE) and Western blotting procedures using precast Mini-PROTEAN TGX gels (Bio-Rad). Equal volumes were loaded on the gels. Antibodies include anti-acetyl p53 K382 (2570, Cell Signaling), anti-acetyl p53 K381 (ab61241, abcam), anti-c-Jun (9165, Cell Signaling), anti-Erk1/2 (9102, Cell Signaling), anti-Fas (ab82419, abcam), anti-JNK (9252, Cell Signaling), anti-p21 (sc-397, Santa Cruz), anti-p53 (MAB1746, R&D systems), anti-Phospho-Erk1/2 T202/Y204 (9255, Cell Signaling), anti-Phospho-JNK T183/Y185 (9255, Cell Signaling), anti-Phospho-c-Jun s63 (2301, Cell Signaling), anti-Phospho-p53 Ser15 (9286, Cell Signaling), anti-PUMA (7467, Cell Signaling), and anti-α-tubulin (T5168, Sigma). Secondary antibodies were obtained from Southern Biotech and Jackson ImmunoResearch. Quantity One software (Bio-Rad) was used for quantification.

### qRT-PCR

All analysis has been conducted on RNA extracts from total retina of mice. Nonaxotomized contralateral retinas were used as control. Total RNA was extracted using Quiazol (Qiagen) protocol. cDNA was produced using Superscript III Reverse Transcriptase (Invitrogen). qRT-PCR analysis was performed on a Light Cycler 480 II (Roche), using LightCycler SYBR Green I Master Mix. Primer sequences are FAS forward 5′-CCCATGCACAGAAGGGAAGGA-3′, reverse 5′-CATGTTCACACGAGGCGCAG-3′; GAPDH forward 5′-CGTCCCGTAGACAAAATGGT-3′, reverse 5′-TTGATGGCAACAATCTCCAC-3′; c-Jun forward 5′-GCCAAGAACTCGGACCTTCTCACGTC-3′, reverse 5′-TGATGTGCCCATTGCTGGACTGGATG-3′; P21 forward 5′-GTGGCCTTGTCGCTGTCTTG-3′, reverse 5′-CCCACGCCTATGGAATGGCT-3′; p53 forward 5′-CCGAAGACTGGATGACTGCCA-3′, reverse 5′-ATGACAGGGGCCATGGAGTG-3′; PERP forward 5′-TTCTTCGCCCTGTGTGGACC-3′, reverse 5′-ATCCGAAGCCATAGGCCCAG-3′; PUMA forward 5′-GGCCTGAGACGCGGCATA-3′, reverse 5′-ATACAGCGGAGGGCATCAGG-3′; TRAIL forward 5′-TTGACCTTTGGCAGGGCTGA-3′, reverse 5′-GCGCTCAGTTGTTTCTGCCA-3′.

### RNA Sequencing

Purified RNA from mouse retinas harvested 2 days after injury in both HDAC1/2 mutant and control mice was analyzed, using the contralateral intact retinas as intact control. The poly(A)-enriched transcriptome were sequenced on Illumina HiSeq2000/2500 (Functional Genomics Center Zürich [FGCZ], Switzerland). Reads were aligned with the STAR (Dobin et al., 2013) aligner with the additional options (–outFilterMatchNmin 30 –outFilterMismatchNmax 5 –outFilterMismatchNoverLmax 0.05 –outFilterMultimapNmax 50). Read alignments were only reported for reads with less than 50 valid alignments. As reference, the mouse genome build and annotation (version 75) from Ensembl (GRCm38) and splice junctions derived from the Ensemble gene annotations were used. Expression counts were computed using the Bioconductor package GenomicRanges (Lawrence et al., 2013). Differential expression was computed using the EdgeR package (Robinson et al., 2010). The reads were normalized using the Trimmed Mean of M values method as described (Dillies et al., 2013). The heat map was created using R (Software version 3.1.0).

### Statistical Analysis

For statistical analyses, Prism 5 (GraphPad Software, Inc.) was applied. Data are shown as the mean ± *SEM*. Statistical significance was determined using one-way analysis of variance test for groups of more than two, and a two-tailed Student’s *t*-test was used for groups of two. Significance was set at *p < .05, **p < .01, and ***p < .001. n represents the number of independent experiments.

## Results

### Generation of Conditional Knockout Mice That Lack HDAC1/2 in RGCs

**Figure 1.**
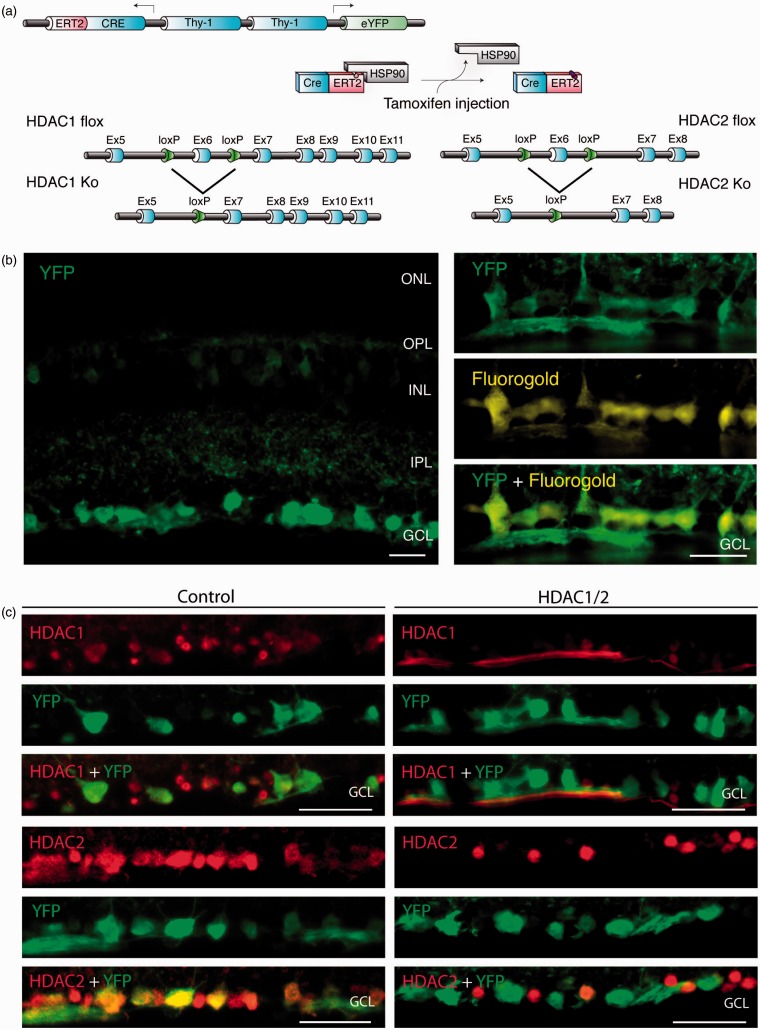
Conditional ablation of HDAC1 and HDAC2 in retinal ganglion cells. (a) The SLICK-H transgenic mouse line uses the *Thy-1* gene regulatory elements to drive coexpression of YFP and a tamoxifen-activatable Cre fusion protein. Schematic map of *Hdac1* and *Hdac2* alleles depicts the location of loxP sequences. Upon Cre-mediated recombination after tamoxifen injection, the genomic region located between the loxP sites is excised, thereby inactivating the conditional *Hdac1* and *Hdac2* alleles. (b) Transverse section of the retina of transgenic mice without tamoxifen injections shows expression of YFP (green) in different retinal layers. ONL = outer nuclear layer; OPL = outer plexiform layer; INL = inner nuclear layer; IPL = inner plexiform layer; GCL = ganglion cell layer. All retrogradely Fluorogold-labeled retinal ganglion cells (yellow) colocalize with YFP (green; right panel). Scale bar: 20 µm (c) Retinal immunofluororescence analysis demonstrates loss of HDAC1/2 in the YFP-expressing cells of the retinal ganglion cell layer after tamoxifen injection in conditional knockout mice. Scale bar: 20 µm.

To address the specific functions of HDAC1/2 in RGCs, we generated tamoxifen-inducible Thy-1-YFP: Thy-1-CreERT2 mice combined with conditional *Hdac1* and *Hdac2* double-floxed alleles (Figure 1(a)). We decided to analyze HDAC1/2 double mutants, since HDAC1 and HDAC2 can often compensate for each other (Montgomery et al., 2007; Ye et al., 2009). In our system, two copies of the Thy1 promoter are used in opposite direction (back-to-back) to simultaneously express CreRT2 and (yellow fluorescent protein) YFP in the same cells. Using retinal immunohistochemistry in transgenic mice without tamoxifen injection, we detected a strong YFP signal in the retinal ganglion cell layer and weaker signals in some cells of the inner nuclear layer. All RGCs, retrogradely labeled with FG, were YFP positive (Figure 1(b)) confirming previous observations using Thy-1-YFP: Thy-1-CreERT2 (SLICK-H) mice (Young et al., 2008). Next, we assessed the efficiency of HDAC1/2 loss in tamoxifen-injected HDAC1/2-double mutants by immunochemistry. Both HDAC1 and HDAC2 were efficiently lost in YFP-positive cells of the ganglion cell layer (Figure 1(c)), establishing the suitability of this genetic model to eliminate HDAC1/2 expression in RGCs.

### Ablation of HDAC1 and HDAC2 in RGCs Promotes Their Survival After Injury

**Figure 2.**
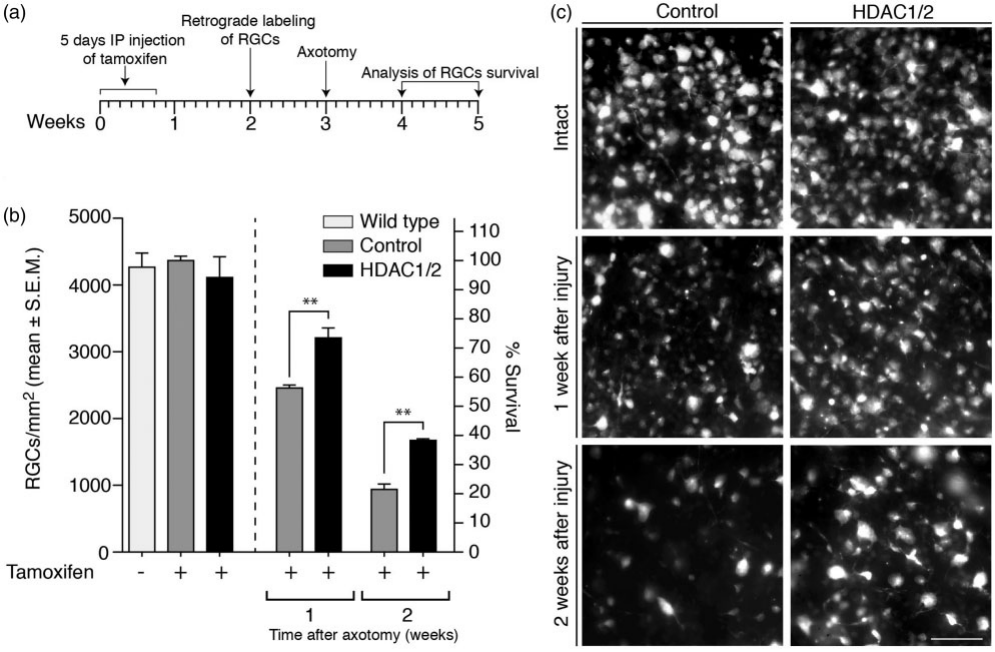
Ablation of HDAC1/2 protects retinal ganglion cells from axotomy-induced death. (a) Outline of the experimental procedure: HDAC1/2 mutants carrying the Thy-1-YFP: Thy-1-CreERT2 allele and littermate controls were injected with tamoxifen for five consecutive days at 2 months of age. Retinal ganglion cells were retrogradely labeled with Fluorogold 9 days after the last tamoxifen injection and subsequently axotomized 1 week later. Retinal ganglion cell survival was assessed by quantification of fluorescent neurons in whole-mounted retinas. (b) Quantitative analysis of retinal ganglion cell survival at 1 and 2 weeks after optic nerve transection in control and HDAC1/2 mutant (labeled HDAC1/2) mice. Error bars represent *SEM* (*n* = 3–4, **p < .01). (c) Fluorescent micrographs of flat-mounted retinae showing representative example of the observed density of Fluorogold-labeled retinal ganglion cells. Scale bar: 50 µm.

Next, we addressed the *in vivo* neuroprotection potential due to specific HDAC1/2 depletion using optic nerve axotomy as injury model in our genetic HDAC1/2 mutants (Figure 2(a))*.* After tamoxifen-induced HDAC1/2 recombination, followed by axotomy, retinae were examined at 1 and 2 weeks postinjury to determine the density of surviving RGCs, visualized using FG labeling. In wild-type uninjured retinae and control (tamoxifen-injected, *Cre*^−^; *Hdac1*^loxP/loxP^ and *Hdac2*^loxP/loxP^) uninjured retinae, the mean density of FG-labeled RGCs was 4,267 ± 211 cells/mm^2^ and 4,365 ± 66 cells/mm^2^, respectively, with no significant difference. Thus, tamoxifen injection itself does not induce RGC death. Furthermore, intact tamoxifen-injected HDAC1/2 mutant retinae, with a cell density of 4,111 ± 309 cells/mm^2^, did not show significantly altered cell death compared with controls. Thus, loss of HDAC1/2 per se does not induce RGC death. However, both at 1 and 2 weeks after injury, HDAC1/2 mutants showed significantly increased RGC survival compared with controls (Figure 2(b) and (c)). One week after injury, 78% ± 0.06 of RGCs survived in HDAC1/2 mutants compared with 56.3% ± 0.02 in the controls (40.7% ± 0.01 compared with 21.5% ± 0.03 after 2 weeks; Figure 2(b)). Together, in agreement with previous studies using pharmacological inhibitors (Pelzel et al., 2010, 2012; Chindasub et al., 2013), these results support HDAC inhibition as a potential therapeutic approach in neuroprotection. Furthermore, HDAC1/2 is identified as specific critical targets.

### Identification of Candidate Pathways Responsible for Neuroprotection due to HDAC1/2 Depletion

**Figure 3.**
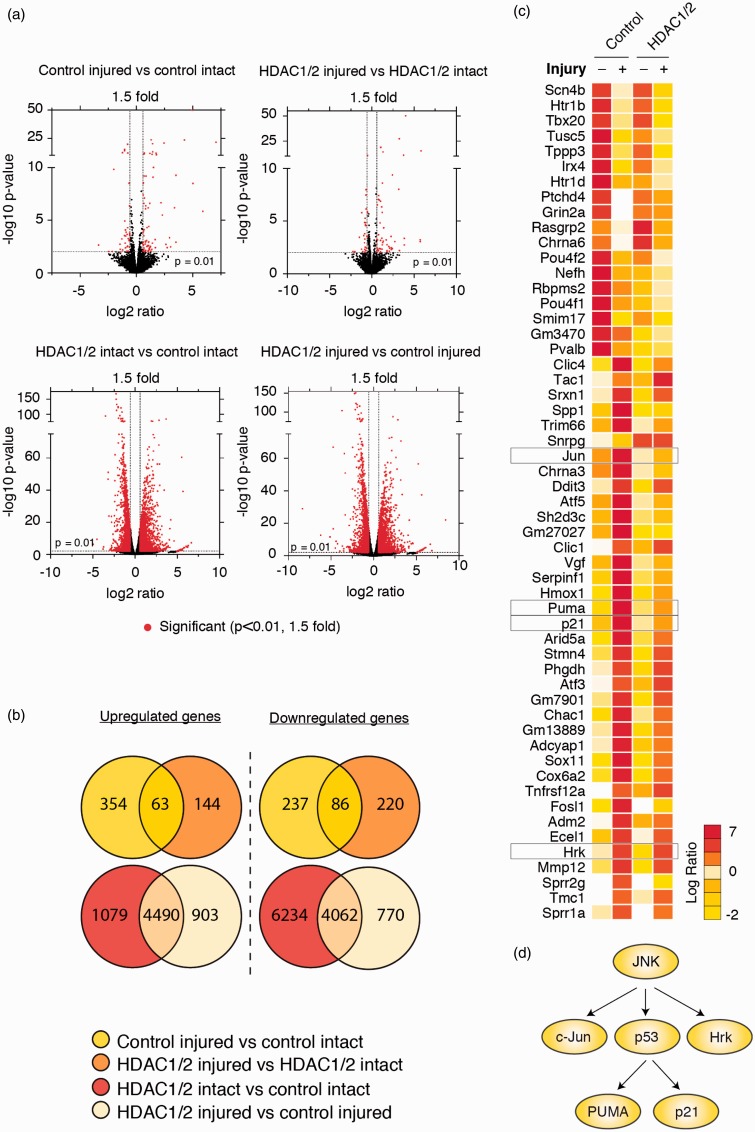
HDAC1/2 ablation causes major changes in transcriptional regulation in the retina. (a) Volcano plots illustrating global transcriptional changes in intact and injured (2 dpi) retinae of HDAC1/2 mutants and control. Each dot represents one gene. The log2 fold change is represented on the *x* axis, and the *y* axis shows the log10 of the *p* value. All genes with a *p* value lower or equal to .01 and a fold change greater than or equal to1.5 are represented in red. (b) Venn diagram showing the total upregulated and downregulated genes in the control and HDAC1/2 mutant retinae, with or without injury (*p* < .05). (c) Heat map depicting changes in expression of the most significantly regulated genes (*p* < .001, fold change ≥ 1.5) in control retinae following optic nerve transection and compared with expression changes between HDAC1/2 mutant intact and injured retinae. (d) The JNK-p53 stress-activated pathway was identified as a major interaction network involved after optic nerve transection.

Early gene silencing and transcriptional dysregulation are hallmarks of models of neurodegeneration, including glaucoma and axonal injury (Ahmed et al., 2004; Yang et al., 2007; Soto et al., 2008), while HDACs can affect transcription at various levels (Nusinzon and Horvath, 2005). Thus, we hypothesised that HDAC1/2 ablation may lead to changes in mRNA levels that may explain the observed neuroprotection phenotype. To address this issue, we analyzed retinal transcriptome profiles of HDAC1/2 mutants and control retinae, with or without injury, by RNAseq. We decided to analyze retinas 2 days postinjury (2 dpi), prior to the onset of evident RGC death that starts around 5 dpi, to avoid major overshadowing secondary effects due to massive cell death (Villegas-Perez et al., 1993; Berkelaar et al., 1994; Lebrun-Julien et al., 2009). While we processed our data, another retinal transcriptome, obtained at 2 days following optic nerve crush, was published (Yasuda et al., 2014). A comparison of those data with our data from injured control retinae revealed, despite the use of slightly different injury models, that nine of their “top 10” upregulated transcripts and five of their “top 10” downregulated transcripts overlap with our top 20 up- and downregulated transcripts (Table 1). Next, we started with a systematic analysis by comparing first intact uninjured retinae from HDAC1/2 mutants to controls (Figure 3(a) and (b)). This analysis revealed a global gene expression shift with 5,569 genes significantly (*p* < .05) upregulated and 10,296 genes downregulated in HDAC1/2 mutants (Figure 3(b)). A similar proportion of activated versus repressed genes has also been observed in cells treated with HDAC inhibitors (Mariadason et al., 2000; Chambers et al., 2003). Given these findings and based on additional comparative analyses of the available four different data sets (controls, HDAC1/2 mutants, both intact and injured; Table 2; Figure 3(a) and (b)), we decided to focus our further evaluations on those genes that were most significantly regulated after injury in controls. We hypothesized that expression changes in these genes have a high likelihood to regulate the ensuing neuronal cell death and survival. In addition, comparing the level of regulation of these genes with that observed between uninjured and injured HDAC1/2 mutants, in combination with a candidate selection approach, may allow us to filter out relevant regulators that are altered due to HDAC1/2 depletion and might be involved in the ensuing neuroprotection effect. Thus, we generated a heat map of the changes of the top up- and downregulated genes using R software with a *p* value under .001 and a fold change ≥1.5 (Figure 3(c)). A similar regulation pattern appeared for changes in controls and in the HDAC1/2 mutants. However, closer inspections, applying the criteria outlined above, identified key components of the proapoptotic JNK-p53 stress-activated pathway, including the JNK target c-Jun and two p53 targets, p21 and PUMA, to be potentially involved in the observed neuroprotection due to HDAC1/2 depletion (Figure 3(c) and (d)).

**Table 1.** Top 20 Upregulated and Downregulated Genes After Injury in Control.

Symbol	Description	Fold change	*p*	The False discovery rate (FDR)
*Upregulated*				
Sprr1a^a^	Small proline-rich protein 1 A	134.74	2.02 E−22	9.08 E−19
Tmc1	Transmembrane channel-like protein 1	60.55	1.43 E−06	6.09 E−04
Sprr2g	Small proline-rich protein 2 G	32.42	3.18 E−09	2.11 E−06
Mmp12^a^	Matrix metallopeptidase 12	31.02	1.01 E−50	1.81 E−46
Hrk	Activator of apoptosis harakiri	19.20	2.83 E−24	1.69 E−20
Ecel1^a^	Endothelin converting enzyme-like 1	11.50	5.33 E−10	4.55 E−07
Adm2	Adrenomedullin 2	10.47	6.65 E−07	3.06 E−04
Fosl1	Fos-like antigen 1	7.76	4.54 E−04	9.15 E−02
Tnfrsf12a^a^	Tumor necrosis factor receptor superfamily, member 12 a	5.51	1.27 E−21	4.57 E−18
Cox6a2	Cytochrome C oxidase subunit via polypeptide 2	4.95	3.26 E−06	1.27 E−03
Sox11^a^	SRY-related HMG-Box 11	4.58	2.18 E−07	1.22 E−04
Adcyap1	Adenylatecyclase activating polypeptide 1	3.41	2.89 E−11	2.88 E−08
Gm13889	Predicted gene 13889	3.37	9.24 E−13	1.18 E−09
Chac1^a^	ChaC, cation transport regulator-like 1 (*E. coli*)	2.88	9.96 E−11	9.41 E−08
Atf3^a^	Activating transcription factor 3	2.72	8.86 E−14	1.59 E−10
Phgdh^a^	3-phosphoglycerate dehydrogenase	2.64	1.27 E−11	1.34 E−08
Stmn4	Stathmin-like 4	2.47	1.98 E−24	1.69 E−20
Arid5a	AT-rich interactive domain 5 A (MRF1-like)	2.31	1.60 E−12	1.92 E−09
Cdkn1a^a^	Cyclin-dependent kinase inhibitor 1 A (P21)	2.28	2.60 E−07	1.37 E−04
Bbc3	BCL2-binding component 3 (PUMA)	2.16	3.85 E−05	1.21 E−02
*Downregulated*				
Scn4b	Sodium channel, voltage-gated, type IV, beta subunit	−4.01	1.44 E−10	1.29 E−07
Htr1b	5-hydroxytryptamine (serotonin) receptor 1B, G protein-coupled	−2.35	3.61 E−13	5.89 E−10
Tbx20	T-Box 20	−2.09	2.89 E−04	6.10 E−02
Tusc5	Tumor suppressor candidate 5	−2.06	7.24 E−15	1.44 E−11
Tppp3^a^	Tubulin polymerization-promoting protein family member 3	−2.05	5.84 E−16	1.31 E−12
Irx4	Iroquois homeobox 4	−2.03	1.17 E−05	4.15 E−03
Htr1d	5-hydroxytryptamine (serotonin) receptor 1D, G protein-coupled	−1.97	8.06 E−05	2.19 E−02
Ptchd4	Patched domain containing 4	−1.94	3.15 E−04	6.50 E−02
Grin2a	Glutamate receptor, ionotropic, N-methyl D-aspartate 2 A	−1.89	1.36 E−04	3.53 E−02
Rasgrp2^a^	Rasguanyl releasing protein 2 (calcium and DAG-regulated)	−1.77	2.23 E−04	5.20 E−02
Chrna6	Cholinergic receptor, nicotinic, alpha 6	−1.72	1.23 E−20	3.16 E−17
Pou4f2^a^	POU domain, class 4, transcription factor 2	−1.70	5.18 E−05	1.50 E−02
Nefh	Neurofilament, heavy polypeptide	−1.69	7.72 E−13	1.07 E−09
Rbpms2	RNA binding protein with multiple splicing 2	−1.58	3.12 E−09	2.11 E−06
Pou4f1	POU domain, class 4, transcription factor 1	−1.54	4.42 E−07	2.20 E−04
Smim17	Small integral membrane protein 17	−1.52	1.89 E−04	4.47 E−02
Gm3470	Predicted gene 3470	−1.51	2.52 E−04	5.66 E−02
Pvalb^a^	Parvalbumin	−1.51	1.64 E−04	4.05 E−02
Kcnd2^a^	Potassium voltage-gated channel, Shal-related family, member2	−1.49	2.61 E−04	5.78 E−02
Rbpms	RNA-binding protein with multiple splicing	−1.42	2.48 E−06	1.01 E−03

*Note.* Differences were considered when FDR was <0.1.

a Overlapping transcript with Yasuda et al. (2014).

**Table 2.** Top 20 Upregulated and Downregulated Genes After Injury in HDAC1/2 Mutants.

Symbol	Description	Fold change	*p*	The False discovery rate (FDR)
*Upregulated*				
Sprr1a	Small proline-rich protein 1 A	56.96	5.60 E−16	2.81 E−12
Mmp12	Matrix metallopeptidase 12	16.02	5.89 E−51	1.18 E−46
Ecel1	Endothelin converting enzyme-like 1	13.51	1.03 E−11	3.27 E−08
Hrk	Activator of apoptosis harakiri	9.63	5.57 E−28	5.58 E−24
Tmc1	Transmembrane channel-like protein 1	9.15	9.47 E−09	1.73 E−05
Synpo2l	Synaptopodin 2-like	5.43	4.15 E−10	1.04 E−06
Piezo2	Piezo-type mechanosensitive ion channel component 2	2.65	4.29 E−05	2.26 E−02
Tnfrsf12a	Tumor necrosis factor receptor superfamily, member 12 a	2.48	7.06 E−15	2.83 E−11
Chac1	ChaC, cation transport regulator-like 1 (E.coli)	2.41	2.53 E−09	5.63 E−06
Prph	Peripherin	2.35	1.55 E−06	1.27 E−03
Pirt	Phosphoinositide-interacting regulator of transient receptor potential channels	2.25	4.50 E−07	4.51 E−04
Stmn4	Stathmin-like 4	2.08	1.24 E−19	8.32 E−16
Angptl2	Angiopoietin-related protein 2	1.98	1.52 E−05	9.53 E−03
Phgdh	3-phosphoglycerate dehydrogenase	1.87	9.90 E−08	1.17 E−04
Atf3	Activating transcription factor 3	1.76	3.78 E−08	5.05 E−05
Arid5a	AT rich interactive domain 5 A (MRF1-like)	1.73	8.65 E−08	1.08 E−04
Cdkn1a	Cyclin-dependent kinase inhibitor 1 A (P21)	1.72	1.99 E−04	8.51 E−02
Gm13889	Predicted gene 13889	1.57	2.92 E−07	3.08 E−04
Tac1	Tachykinin, precursor 1	1.47	2.57 E−05	1.43 E−02
Trim66	Tripartite motif containing 66	1.47	6.20 E−06	4.29 E−03
*Downregulated*				
Scn4b	Sodium channel, voltage-gated, type IV, beta subunit	−2.28	1.58 E−06	1.27 E−03
Gm15564	Predicted gene 15564	−1.94	2.41 E−04	9.66 E−02
Irx4	Iroquois homeobox 4	−1.83	1.34 E−05	8.96 E−03
Tbx20	T-Box 20	−1.78	1.61 E−04	7.32 E−02
Pou4f2	POU domain, class 4, transcription factor 2	−1.72	5.42 E−06	3.89 E−03
Rasgrp2	Iroquois homeobox 4	−1.70	1.39 E−05	8.98 E−03
Rprm	Reprimo, TP53 dependent G2 arrest mediator candidate	−1.69	2.36 E−05	1.40 E−02
Tusc5	Tumor suppressor candidate 5	−1.62	3.35 E−08	4.80 E−05
Fxyd7	FXYD domain containing ion transport regulator 7	−1.61	2.63 E−05	1.43 E−02
Htr1b	5-hydroxytryptamine (serotonin) receptor 1B, G protein-coupled	−1.59	7.09 E−07	6.77 E−04
Tppp3	Tubulin polymerization-promoting protein family member 3	−1.59	1.06 E−08	1.76 E−05
Rbpms2	RNA binding protein with multiple splicing 2	−1.44	1.25 E−06	1.14 E−03
Chrna6	Cholinergic receptor, nicotinic, alpha 6 (neuronal)	−1.41	1.14 E−11	3.27 E−08
Sncg	Synuclein, gamma	−1.41	1.57 E−06	1.27 E−03
Calb2	Calbindin 2	−1.34	3.31 E−06	2.46 E−03
Nefh	Neurofilament, heavy peptide	−1.31	1.19 E−04	5.80 E−02
Rnf208	Ring finger protein 208	−1.30	2.37 E−04	9.66 E−02
Tubb4a	Tubulin, beta 4 A class IVa	−1.29	2.07 E−06	1.60 E−03
Rbpms	RNA binding protein with multiple splicing	−1.29	2.22 E−04	9.28 E−02
Nat8l	N-acethyltransferase 8-like (GCN5-related, putative)	−1.27	1.95 E−04	8.51 E−02

*Note.* ^a^Differences were considered when FDR was < 0.1.

### Comparable Activation of the JNK Pathway in Control and HDAC1/2-Ablated Retinae After Injury

**Figure 4.**
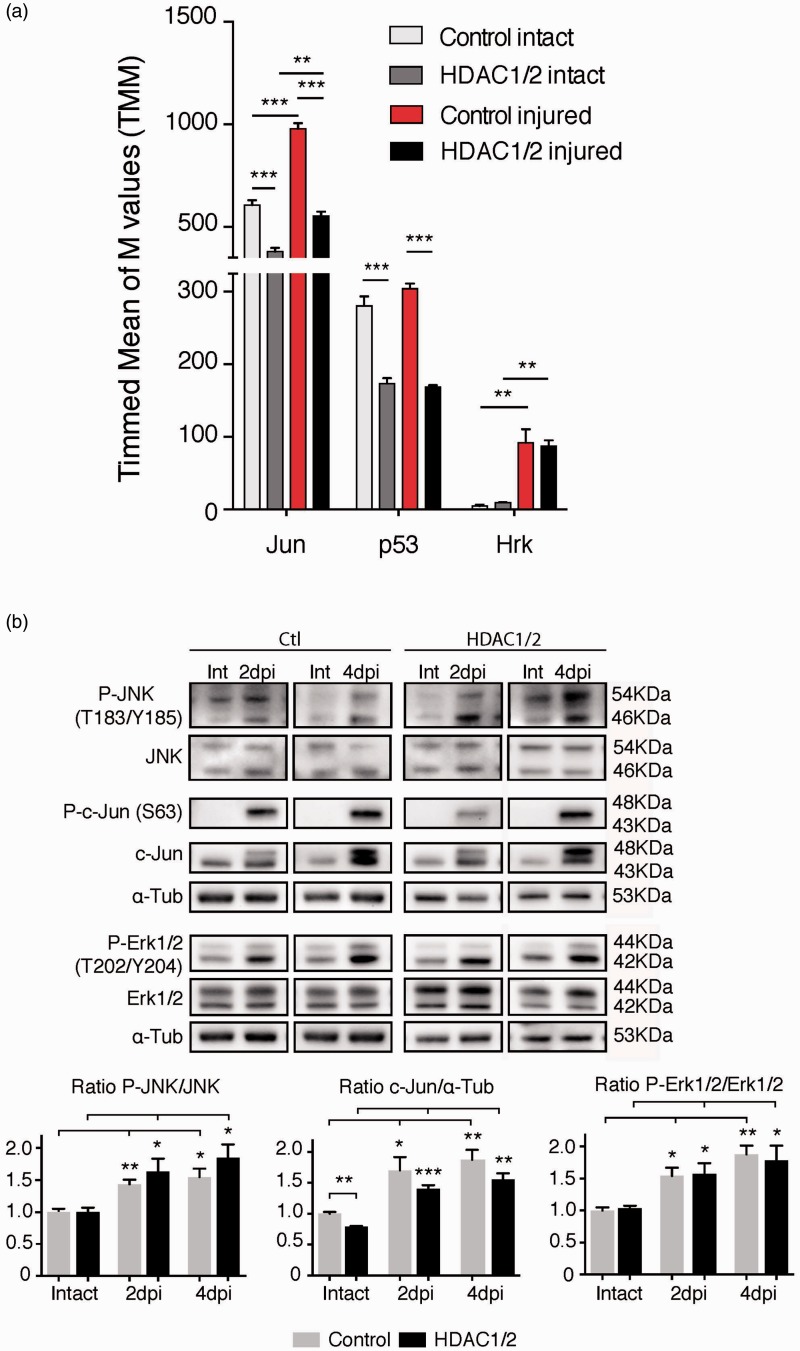
MAP kinase pathways are activated comparably in HDAC1/2 mutants and controls following injury. (a) RNA-seq analysis of the JNK targets Jun, p53, and Hrk. Error bars indicate *SEM* (*n* = 3, *p < .05, **p < .01, ***p < .001). (b) Retinae lysates of control and HDAC1/2 mutants, with or without injury, were analyzed by immunoblotting at 2 dpi and 4 dpi to assess activation of MAP kinase pathways (JNK, c-jun, and Erk1/2). Alpha-tubulin was used as control. Error bars indicate *SEM* (*n* = 3, *p < .05, **p < .01, ***p < .001). Int = intact, not injured. Ctl = control.

JNK is a member of the mitogen-activated protein kinase family (also known as the stress-activated protein kinases or SAPKs) that is activated in response to a variety of cellular stresses. Sustained activation contributes to cell death through different targets including c-Jun (Whitfield et al., 2001; Ma et al., 2007, p. 53; Fuchs et al., 1998a, 1998b) and Hrk (Chang et al., 2013). Previous studies have demonstrated that JNK signaling and its target transcription factor c-Jun are activated in RGCs following optic nerve injury. Furthermore, double deficiency for *Jnk2* and *Jnk3* strongly protects mouse RGCs from injury (Fernandes et al., 2012, 2013). Thus, we assessed the role of the JNK pathway in our model. At the transcriptional level, RNA-seq analysis showed that the JNK target c-Jun was increased in both HDAC1/2 mutants and controls after injury, but the basal level and the upregulation were lower in the mutant (Figures 3(c) and 4(a)). As expected, further analysis of the RNA-seq data of injured control retinae revealed also upregulation of harakiri (Hrk), a regulator of cell death that belongs to the family of proapoptotic BH3-only proteins that are mainly found in the nervous system tissue (Wakabayashi et al., 2002; Figures 3(c) and 4(a)). However, mutant injured retinae showed a comparable upregulation to controls, in line with a recent study concluding that Hrk is not the main effector for RGC death after injury (Fernandes et al., 2013; Figures 3(c) and 4(a)). p53 mRNA levels did not change after injury. However, they were significantly lower in both intact and axotomized mutant retinas compared with controls (Figure 4(a)).

Next, we examined the ability of HDAC1/2-ablated RGCs to activate the JNK pathway following injury. Thus, we analyzed retinal homogenates of control and HDAC1/2 mutant mice after 2 dpi and 4 dpi. In both HDAC1/2 mutants and controls, the injury triggered comparable activation by phosphorylation of JNK and its target c-Jun, sustained at 4 dpi in both cases (Figure 4(b)). Consistent with the observations at the mRNA level, the basal protein level of c-Jun was significantly lower in the intact mutant retinae but increased after injury. Since Erk1/2, another mitogen-activated protein kinase, is known to protect RGCs upon sustained activation (Cheng et al., 2002; Zhou et al., 2005), we tested also the ability of HDAC1/2-ablated RGCs to activate this pathway. We observed similar activation of Erk1/2 at 2 dpi and 4 dpi both in the mutant and control retinae (Figure 4(b)). Taken together, these data indicate that HDAC1/2 mutants retain largely the ability to activate the MAP kinase pathways.

### Transcription of PUMA and p21 Is Impaired in HDAC1/2-Ablated and Axotomized RGCs

**Figure 5.**
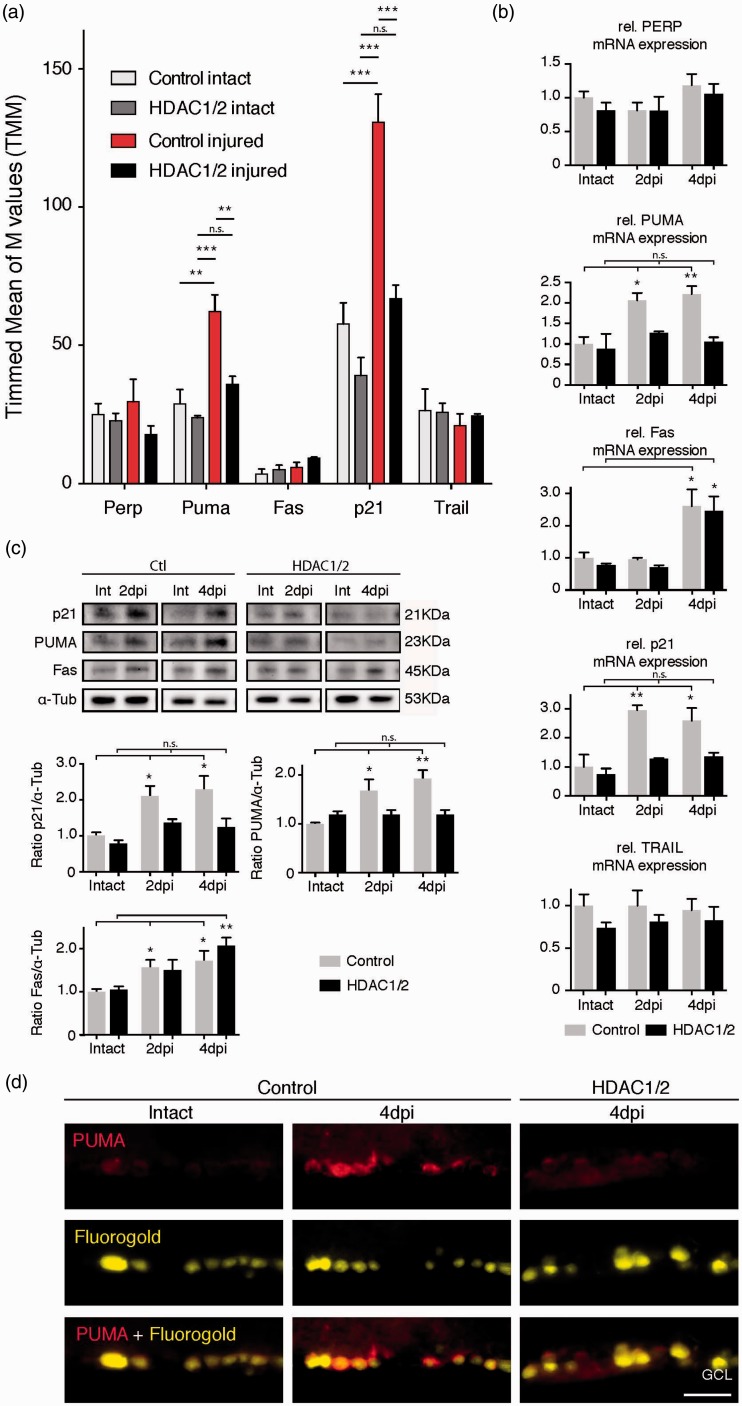
Ablation of HDAC1/2 reduces p53-dependent transcription of PUMA and p21 following injury. (a) RNA-seq analysis of p53 targets in HDAC1/2-ablated retinae. Error bars indicate *SEM* (*n* = 3, **p < .01, ***p < .001). (b) Confirmation of RNA-seq analysis by qRT-PCR, with extension to 4 dpi. Note: Fas mRNA upregulation at 4 dpi but not at 2 dpi. Error bars indicate *SEM* (*n* = 3, *p < .05, **p < .01). (c) Western blot analysis of main p53 targets revealed patterns comparable to those observed at the mRNA level. Error bars indicate *SEM* (*n* = 3, *p < .05, **p < .01). (d) Retinal immunohistochemistry showing increased expression of the proapoptotic protein PUMA in retinal ganglion cells following injury (4 dpi) in the control and reduced signal in HDAC1/2 mutant. Scale bar: 250 µm.

We decided to follow-up on the observed lower p53 levels in HDAC1/2 mutants compared with controls (Figure 4(a)) since p53 is a major regulator of apoptosis (Elmore, 2007). More specifically, p53 acts as a transcription factor in intrinsic and extrinsic apoptosis signaling pathways by initiating transcription of mRNAs that encodeproapoptotic proteins such as PUMA, Bax, Fas/CD95, PERP, and TRAIL, as well as cell cycle arrest proteins including p21/Cdkn1a (Attardi et al., 2000; Beckerman and Prives, 2010). Previous studies in a rat model of optic nerve injury have suggested that p53 acts through PUMA and Fas activation in apoptotic RGCs. Consistently, RGCs in p53 null mice are partially resistant to axotomy-induced death (Wilson et al., 2013, 2014). To obtain an overview, we analyzed the regulation of p53 targets in our mouse models. Inspection of the RNA-seq data revealed a strong increase of two main p53 targets, p21 and PUMA after injury in controls, but not in HDAC1/2 mutants (Figure 5(a)). Other p53 targets including Perp, Fas, and Trail showed no significant differences (Figure 5(a)). We validated the RNA-seq data by qRT-PCR, including a second time point at 4 dpi (Figure 5(b)). Of interest, Fas mRNA levels were not changed at 2 dpi, but increased both in HDAC1/2 mutants and controls at 4 dpi (Figure 5(b)). To corroborate the data at the protein level, we performed Western blot analysis of retina samples. As on the mRNA level, PUMA and p21were increased in controls at both time points after injury, while there were no significant changes in the HDAC1/2 mutants (Figure 5(c)). As expected, Fas was upregulated in both HDAC1/2 mutants and controls at 4 dpi and also significantly at 2 dpi in controls with a trend in mutants (Figure 5(c)). Since we used whole retina extracts for these analyses, contributions by other cell types may mask partially signal differences. Thus, we analyzed PUMA expression in RGCs at the cellular level using immunohistochemistry. The injury induced a robust increase of PUMA in FG-labeled RGCs in controls at 4 dpi, whereas HDAC1/2-ablated RGCs showed no difference compared with control intact retinae (Figure 5(d)). Collectively, these data indicate that correct selective activation of p21 and the proapoptotic target PUMA fails as an early response after optic nerve injury in HDAC1/2 mutants, likely mediated by altered p53.

### Increased Acetylation at K381 Reduces the Proapoptotic Activity of p53 in Axotomized HDAC1/2 Mutants

**Figure 6.**
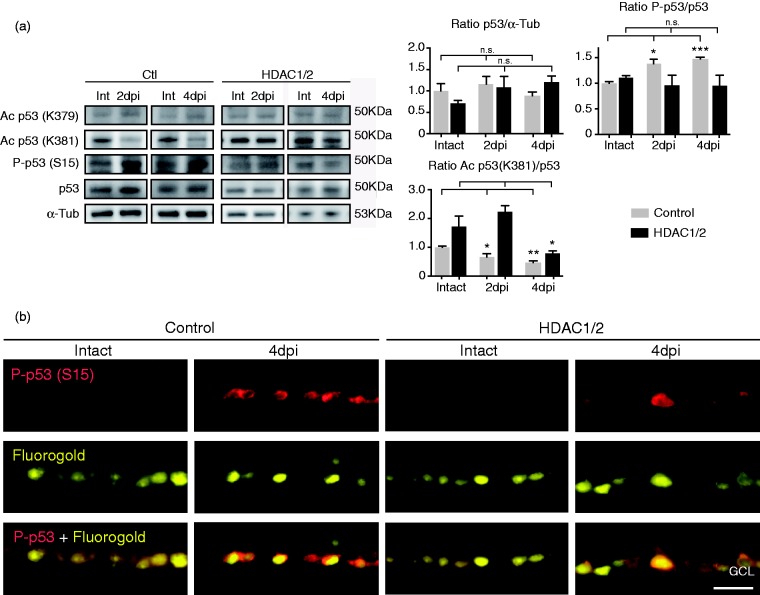
Aberrant posttranslational p53 modifications in HDAC1/2 mutant retinae following injury. (a) Western blot analysis of different posttranslational modifications of p53 in controls and HDAC1/2 mutants following optic nerve transection (2 dpi and 4 dpi). Error bars indicate *SEM* (*n* = 3, *p < .05, **p < .01,***p < .001). (b) Retinal immunohistochemistry revealed increased p53 phosphorylation at Ser15 (S15) in axotomized retinal ganglion cells (4 dpi) in control retina. Only few immune-positive cells are present in HDAC1/2-ablated retinal ganglion cells. Scale bar: 250 µm.

Posttranslational modifications of p53 are essential for its activation and appropriate acetylation of p53 on key lysine residues of the C-terminal region is needed to induce full transcription of its target genes (Gu and Roeder, 1997; Knights et al., 2006; Zhao et al., 2006). A recent *in vitro* study suggests that acetylation of p53 specifically on lysines K381 and K382 (the human equivalent of mouse K379) inhibits its ability to activate transcription of PUMA in cortical neurons (Brochier et al., 2013). Following a related rationale, we analyzed posttranslational modifications of p53 in HDAC1/2-ablated RGCs compared with control at 2 dpi and 4 dpi. Even though the amount of p53 mRNA was decreased in HDAC1/2 mutant retinae compared with controls, both with and without injury (Figure 4(a) and (b)), we detected no significant changes at the protein level (Figure 6(a)). However, in control retinae at both time points analyzed after injury, we found increased phosphorylation of p53 at serine (S15), which is correlated with the ability of p53 to trigger apoptosis (Olsson et al., 2007; Boehme and Blattner, 2009; Figure 6(a) and (b)). These data are consistent with findings in the rat model of axotomy (Wilson et al., 2013). Furthermore, this p53 activation is in line with the observed parallel increase of PUMA expression (Figure 5(d)). HDAC1/2-ablated RGCs showed only sparse p53 phospho-S15 signals by immunohistochemistry and no detectable change by Western blot analysis (Figure 6(a) and (b)). Next, we analyzed the acetylation pattern of p53 on the two key lysine residues, K381 and K379. No significant differences comparing HDAC1/2 mutant and control retinae were detected on residue K379 with or without axotomy (Figure 6(a)). However, we found that axotomy triggered significant deacetylation of K381 in control retinae, both at 2 dpi and 4 dpi. HDAC1/2 ablation in RGCs revealed a trend of increased acetylation early after injury (2 dpi), in line with impairment of p53 activation and transcription of its proapoptotic target PUMA. At 4 dpi, we discovered that K381 acetylation was significantly reduced in HDAC1/2-ablated RGCs by a yet unknown mechanism.

## Discussion

The present study explored the functions of HDACs during neurodegeneration. More specifically, we investigated the role of HDAC1/2 in the regulation of adult RGC survival *in vivo* using specific and accurate genetic tools. Our study supports four major conclusions. First, combined specific ablation of *HDAC1* and *HDAC2* in RGCs promotes neuroprotection following axonal injury *in vivo*. Second, the JNK-p53-PUMA pathway is activated following injury in axotomized mouse RGCs. In HDAC1/2-ablated RGCs, p53 activation and the transcription of its proapoptotic target PUMA are significantly impaired. Third, this inability of HDAC1/2 mutants to activate p53 correlates with the abnormal acetylation profile of p53 on K381. Fourth, HDAC1/2 inhibition provides a specific target for the development of neuroprotective therapies.

HDACs inhibitors have been shown to be neuroprotective in different neurodegenerative models both *in vivo* and *in vitro*, but the exact mechanisms of action are still poorly understood (Chuang et al., 2009). Since HDACs act on a wide variety of substrates including histones, transcription factors, and cytosolic proteins, it is of paramount importance to evaluate their function in each specific cell type prior to the elaboration of new therapeutic strategies. Therefore, to unravel the molecular mechanisms that underlie the neuroprotective potential of HDAC inhibition, we created conditional knockout mice for HDAC1 and HDAC2 in RGCs (Young et al., 2008). Here, we show that efficient genetic ablation of both *HDAC1* and *HDAC2 in vivo* promotes RGC survival following injury. These results are consistent with recent studies using injection of the HDACs inhibitor ms-275, active against HDAC1 and 3, or trichostatin A, an inhibitor of Class I and II HDACs, in an optic nerve injury model (Gaub et al., 2011; Chindasub et al., 2013). Taken together, those studies showed that an overall increase in cellular acetylation enhances survival of RGCs but did not provide definitive answers on specificity issues. Others, also using trichostatin A, concluded that histone deacetylation mainly by HDAC3 plays an important role in the early gene silencing response of apoptotic RGCs (Pelzel et al., 2010). Partially following this rationale, we performed an RNA-seq analysis of our retina samples to investigate the early response following axonal injury in the control compared with the HDAC1/2-ablated retinae. Another group recently published the retinal transcriptome at 2 days after optic nerve crush injury; pathway analysis revealed a cell death and survival response early after injury (Yasuda et al., 2014). This study showed that ER stress was the major response triggered, followed by other pathway alterations including RGCs marker downregulation and immune response activation. Our RNA-seq analysis in the axotomy model revealed similar changes, sharing most the top upregulated and downregulated targets in control retinae (Yasuda et al., 2014). On the other hand, the mutant intact retinae revealed that HDAC1/2 ablation per se induced already major modifications of the transcription profile. Interestingly, more genes were downregulated rather than upregulated, confirming that HDAC function is more complex than only inducting gene repression. We cannot rule out that this gene expression shift played a role in the neuroprotection effect observed after injury. However, despite this global transcriptome difference, the most significantly upregulated and downregulated genes triggered by the injury shared a similar pattern in the mutant retinae compared with controls. Indeed, we found that the stress-activated protein kinase JNK, and its targets are likely to play important roles in the answer following injury. Our data corroborate previous observations showing that JNK and c-Jun are highly activated in injured RGCs, and that deletion of *Jnk2* and *Jnk3* promotes RGC survival (Fernandes et al., 2012). Here, we confirmed that JNK and its target c-Jun are activated in axotomized RGCs in both the HDAC1/2 mutant and control retinae. In addition, the mRNA level of the proapoptotic target Hrk was also upregulated confirming previous observations in axotomized RGCs (Wakabayashi et al., 2002). However, these targets could not explain the neuroprotective phenotype observed in the HDAC1/2 mutants. In stressed cells, JNK signaling can stabilize and activate the proapoptotic transcription factor p53 (Fuchs et al., 1998b). We detected activation of p53 in controls after injury, resulting in increases at both the transcription and protein level of two of its targets, p21 and PUMA. In the HDAC1/2 mutant retinae, neither p53 nor its targets were activated, leading us to the conclusion that p53 may be responsible for the neuroprotection effect observed.

The transcription factor p53 regulates neuronal apoptosis during nervous system development, as well as in neurodegenerative conditions (Miller et al., 2000; Morrison and Kinoshita, 2000). In the developing retina, p53 is abundantly expressed and eventually downregulated during maturation (O’Connor et al., 2008). Wilson et al. (2013) recently demonstrated that axotomized rat RGCs die in a p53-dependent manner through activation of the proapoptotic targets PUMA and Fas. They showed that RGCs abundantly express ASPP1 and ASPP2, the apoptosis-stimulating proteins of p53, and that selective knockdown of those targets leads to RGC survival after injury in rats. In addition, siRNA against PUMA or Fas conferred neuroprotection in that study. Consistent with these findings, our data in mice also showed an increase in Fas and PUMA in control axotomized RGCs, but only the increase in PUMA was impaired in HDAC1/2 mutants. These data corroborate the critical role played by PUMA in injury-induced RGC loss (Wilson et al., 2013). Transcription of Fas through p53 was not altered in the HDAC1/2 mutant retinae, which probably contributed to death of RGCs. Further experiments may help to explain why transcription of Fas remained in HDAC1/2 mutants, although posttranscriptional modifications of p53 is well known to differentially activate Fas following DNA damages (Kobayashi et al., 1998). Of note in this context, Fas and p53 have been reported to signal apoptosis independently, depending of the cell type (O’Connor et al., 2000).

Our data demonstrate that p53 activation is impaired in HDAC1/2-ablated retinae. Regulation of p53 function is exerted via a plethora of posttranslational modifications such as phosphorylation, acetylation, and methylation (Olsson et al., 2007; Boehme and Blattner, 2009). As one of the first nonhistone proteins that was shown to be acetylated by histone acetyltranferase, p53 acetylation on the C-terminus is commonly recognised as a stabilizing and activating modification (Gu and Roeder, 1997; Boehme and Blattner, 2009). However, more recent studies suggested that specific acetylation of p53 at different sites can trigger or inhibit transcription of different target genes in different cell type (Knights et al., 2006; Brochier et al., 2013). To investigate whether changes in p53 acetylation status in the HDAC1/2 mutants might account for the inability to trigger PUMA and p21 expression, we examined p53 acetylation at K381 and K382. In cultured cortical neurons, specific acetylation of p53 at K381 and K382 resulting from HDAC inhibition prevented transcription of PUMA and p21 and lead to neuronal survival (Brochier et al., 2013). The same study showed that acetylation on the same site can trigger the opposite effect in cancer cells, leading the authors to conclude that specific acetylation of p53 in different cell type can trigger different effects. We detected parallel deacetylation at K381 and increase phosphorylation at S15 of p53 in axotomized RGCs in control mice, pointing toward the necessity of deacetylation of this lysine residue for proper p53 activation. In contrast, in the HDAC1/2 mutants, K381 remained highly acetylated after injury and no significantly increased phosphorylation of p53 was observed in RGCs, suggesting that HDAC1/2 is likely to play a role in the deacetylation/activation of p53 at K381 in RCGs. Further studies will be required to determine the extent of direct connections between temporal and spatial regulation of HDAC1/2 activity, degree of p53 acetylation, PUMA expression, and RGC survival. Also, additional analysis of other acetylation sites might provide a better understanding of the p53 activation process in different neuronal populations *in vivo*, but other possibilities may also be raised. For example, specific acetylation of p53 could intervene in the recruitment of neuron-specific cofactors, which could in turn modulate the affinity of p53 for specific promoters (Sims and Reinberg, 2008). Furthermore, since lysine residues that can be subjected to acetylation are also targeted by methyl-transferases, with opposing effects on p53 function, it will be of interest to analyse the methylation profile of p53. For example, methylation at K372 can enhance p53-dependent transcription (Chuikov et al., 2004), whereas methylation at K370 mediates repression of transcriptional activity (Huang et al., 2006).

In summary, we have generated an efficient mouse model for conditional recombination in adult mouse RGCs. By contributing to the molecular understanding of how specific ablation of HDAC1/2 is able to promote adult RGC survival after injury *in vivo*, our study provides new insight into how HDAC inhibition can play a neuroprotective function. Understanding how the balance of acetylation/deacetylation leads to neuroprotection is essential for the development of therapeutic strategies for neurodegenerative diseases. Our data suggest that targeting specific acetylation of p53 at K381 represents a relevant target.
